# A Novel Dataset for Gait Activity Recognition in Real-World Environments

**DOI:** 10.3390/s26030833

**Published:** 2026-01-27

**Authors:** John C. Mitchell, Abbas A. Dehghani-Sanij, Shengquan Xie, Rory J. O’Connor

**Affiliations:** 1 School of Mechanical Engineering, University of Leeds, Leeds LS2 9JT, UK; john.mitchell@sheffield.ac.uk; 2 School of Computer Science, University of Sheffield, Sheffield S10 2TN, UK; 3 School of Electronic and Electrical Engineering, University of Leeds, Leeds LS2 9JT, UK; s.q.xie@leeds.ac.uk; 4 Academic Department of Rehabilitation Medicine, University of Leeds, Leeds LS1 3EX, UK; 5 NIHR Devices for Dignity, Sheffield Teaching Hospitals NHS Trust, Sheffield S10 2JF, UK

**Keywords:** force sensors, human activity recognition, inertial sensors, real environments, sensor systems, terrain, wearable sensors, wireless sensor networks

## Abstract

Falls are a prominent issue in society and the second leading cause of unintentional death globally. Traditional gait analysis is a process that can aid in identifying factors that increase a person’s risk of falling through determining their gait parameters in a controlled environment. Advances in wearable sensor technology and analytical methods such as deep learning can enable remote gait analysis, increasing the quality of the collected data, standardizing the process between centers, and automating aspects of the analysis. Real-world gait analysis requires two problems to be solved: high-accuracy Human Activity Recognition (HAR) and high-accuracy terrain classification. High accuracy HAR has been achieved through the application of powerful novel classification techniques to various HAR datasets; however, terrain classification cannot be approached in this way due to a lack of suitable datasets. In this study, we present the Context-Aware Human Activity Recognition (CAHAR) dataset: the first activity- and terrain-labeled dataset that targets a full range of indoor and outdoor terrains, along with the common gait activities associated with them. Data were captured using Inertial Measurement Units (IMUs), Force-Sensing Resistor (FSR) insoles, color sensors, and LiDARs from 20 healthy participants. With this dataset, researchers can develop new classification models that are capable of both HAR and terrain identification to progress the capabilities of wearable sensors towards remote gait analysis.

## 1. Introduction

Falling is the second leading cause of unintentional death globally [1]. According to the World Health Organization (WHO), 684,000 falls are fatal each year, while a further 37.3 million require medical attention, with certain groups being at increased risk of falls due to age and various cognitive or physical conditions [1,2,3]. Gait analysis is a useful approach in healthcare settings to monitor changes in a person’s gait and identify factors that may increase the risk of falling [4]. However, clinical gait analysis has some limiting factors, with studies reporting issues with poor reproducibility and accuracy between centers [4,5]. Modern research has produced sensor systems that are capable of performing accurate reproducible gait analysis whilst enabling additional benefits, such as low power costs, portability, and Internet of Things (IoT) integration [6,7]. These developments allow gait data to be captured using a standardized approach in real-world scenarios, which can then be analyzed remotely or automatically to increase the quality of the data and reduce the time and resources required from healthcare systems.

While these advances create new opportunities for real-world remote gait analysis, they also present new challenges and problems that must be solved. One such problem is determining the activity a patient is performing when unobserved. HAR studies typically aim to use powerful classification techniques and novel deep learning architectures to process real-time data from various open HAR datasets with high reported performance metrics [8,9]. However, another issue that is largely unaddressed in the literature is that of variation in terrain as gait parameters such as footpath and gait variability have been shown to be dependent on the terrain underfoot [10,11]. If patients are to be equipped with a sensor system to wear outside the laboratory whilst gait data is collected, accurate terrain recognition is crucial to provide healthcare specialists with the appropriate context about the data that is being analyzed. This highlights a need for real-terrain gait datasets that enable researchers to extend their successes in activity classification to classifying both activity and terrain so that these systems can be reliably adopted into healthcare settings.

### 1.1. Real-Terrain Activity Recognition Datasets

In recent years, some real-terrain gait datasets using wearable sensors have been proposed. In 2020, Luo et al. [12] collected a dataset from 30 subjects performing five activities on seven unique terrains: (1) walking on a flat paved terrain; (2) ascending concrete stairs; (3) descending concrete stairs; (4) walking up a concrete slope; (5) walking down a concrete slope; (6) walking on grass; (7) walking on the left bank of a paved walkway; (8) walking on the right bank of a paved walkway; and (9) walking on uneven stone bricks. The participants performed these activities whilst wearing six IMUs on the dorsal forearm of the wrist; both anterior thighs; 5 cm proximally from the bony processes of both ankles; and on the lower back at the posterior of the L5/S1 joint [12]. Similarly, Losing and Hasenjäger [13] proposed a dataset featuring 20 subjects performing nine activities across five unique outdoor terrains: (1) walking on a level area; (2) walking up a flat ramp; (3) walking down a flat ramp; (4) walking up a steep ramp; (5) walking down a steep ramp; (6) walking upstairs; (7) walking downstairs; (8) walking on a lay-by with a curb; (9) walking around a 90-degree bend; (10) stepping up a curb in a lay-by; (11) stepping down a curb in a lay-by; and (12) turning 180 degrees. The participants performed these activities whilst wearing FSR insoles in each shoe with eight FSRs each, an eye tracker, and 17 nodes containing both an IMU and barometer on the head, sternum, sacrum, shoulders, upper arms, forearms, hands, upper legs, lower legs, and feet [13]. Whilst providing novel insights into real-terrain gait and the potential for real-environment HAR, these datasets only consider outdoor terrains and do not offer the full range of activities that someone would encounter in a regular day, such as standing or sitting down.

### 1.2. Contributions

This study contributes to the field of real-environment HAR through the collection of a novel activity-and-terrain-labeled HAR dataset, dubbed the CAHAR dataset, which features common walking activities performed on a wide range of terrains, both indoors and outdoors, which are representative of those encountered in a person’s daily life. To collect this dataset, a novel sensor system is designed, which combines sensor types known to be capable of activity classification, such as IMUs and FSR insoles, with lesser-explored sensor types like LiDAR and color sensors. This structured environment-labeled dataset will enable the development of machine learning systems that are capable of detecting terrains in real time, which, when deployed in HAR sensor systems, will allow for the detection of both terrain and activity in free-living contexts. Although this dataset itself does not contain a period of free-living data collection, such a dataset is required to construct supervised classification models that are capable of capturing the context, consisting of both terrain and activity recognition, of collected gait data.

## 2. Methods

### 2.1. The Sensor System

**Figure 1:**
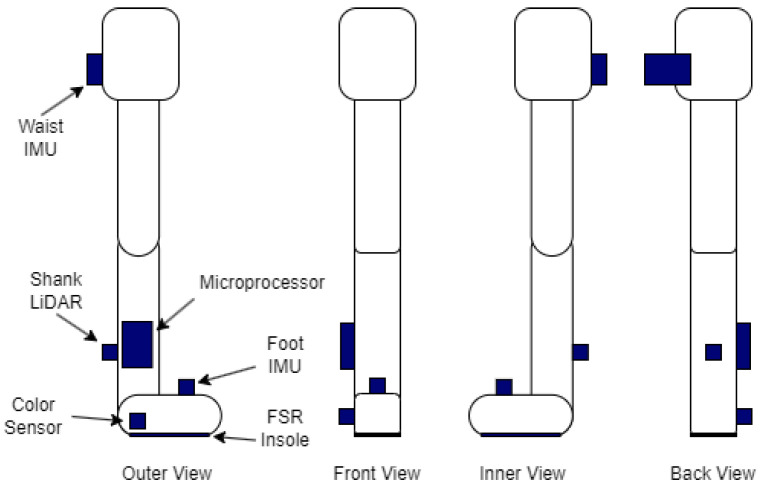
The position of each sensor and the microprocessor on the right leg from various angles.

There are numerous datasets for HAR with wearable sensors, each collected with a unique sensor system. Typically, these datasets make use of inertial sensors, which have proven effective in achieving high-accuracy HAR [7,12,13,14,15,16,17]. Additionally, existing gait analysis makes use of force plates, which can be used to determine the heel-strike and toe-off stages of the gait cycle, along with spatio-temporal gait parameters such as Center of Pressure (CoP) [4,6]. Due to the prominence of these sensors in the literature, IMUs and force-sensing insoles are included in the proposed sensor system, alongside 2 new sensor types that are theorized to assist with terrain classification. These sensors are a color sensor, which will aid in the detection of terrains with consistent colors such as grass, and a LiDAR that can be used to build noise profiles of the ground to differentiate between smooth and rough terrains. A diagram of the sensor system can be found in Figure 1.

An InvenSense ICM-20948 9-DoF IMU (TDK InvenSense, San Jose, CA, USA) is positioned on the waist of the subject and on the top of each foot. This IMU was chosen due to the inclusion of a magnetometer for additional data dimensionality, low power requirements, and the inclusion of a digital motion processor to reduce drift [18]. While IMUs have been shown to be effective in HAR studies [7,12,13,14,15,16,17], they have also demonstrated viability for use in terrain classification contexts [19,20,21] and should provide rich data for both aspects of this dataset.

**Figure 2:**
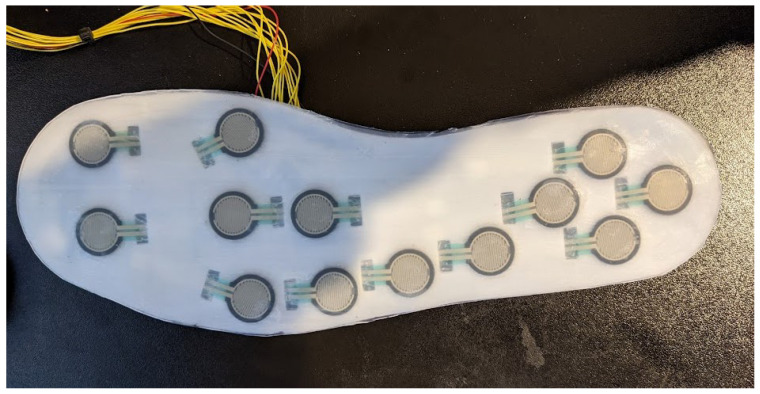
The top side of the FSR insole, showing the positioning of the 13 sensors.

The FSR insoles consist of 13 Interlink 402 FSRs (Interlink Electronics, Inc., Fremont, CA, USA) each, the layout of which can be seen in Figure 2. These insoles consist of a 0.6 mm thick layer that is 3D-printed using Polylactic Acid (PLA) at a chosen shoe size. The FSRs are attached to the insole via a silicone-based adhesive, which is then covered by a 1 mm thick layer of silicone. A layer of flexible prototyping board is adhered to the underside of the PLA layer, to which the FSRs are soldered. Simple potential divider circuits are used to obtain voltage readings from the changes in resistance that the FSRs exhibit, and the location of these circuits changes between 2 versions of the insoles. Version 1 insoles feature the potential divider circuits on the base of the insole, with the advantage of reducing the number of wires from the insole to 15:13 data channels along with positive and ground wires. However, a second version of the insole was later fabricated, which moved the circuitry away from the base of the insole and reduced manufacturing times. Both versions feature an additional 1 mm layer of silicone beneath the circuitry layer, and a multiplexer is used to reduce the number of inputs from 13 to 5 before connecting to the microprocessors. These insoles feature an increased number of sensors when compared with those used in the context-aware HAR dataset proposed by Losing and Hasenjäger [13], with the hope of increasing the performance of these sensors and increasing their contribution to accurate terrain and activity classification.

A TCS34725 color sensor (DFRobot, Shanghai, China) is positioned on the outside of the heel, facing the ground. This sensor provides the red, green, and blue color components of the ground directly underfoot, as well as ambient light levels. This allows analytical methods to easily identify constant-color terrains such as grass and paving slabs, along with helping to determine if the terrain is indoor or outdoor. As the TCS34725 monitors the color of the reflections when shining a light on an object [22], the operating range is low, resulting in the need for the sensor to be placed on the side of the foot and as low to the ground as possible.

To address the coarseness of the terrain underfoot, a VL53L1X Time-of-Flight (ToF) LiDAR (STMicroelectronics, Geneva, Switzerland) is placed at the back of the shank, pointing towards the ground. By mounting the sensor to a fixed point on the shank, the distance between this segment and a point on the ground underfoot can be monitored during gait. As a ToF sensor, the LiDAR is susceptible to variations in leg motion and orientation, the former of which can be exploited for gait activity classification, while the latter can be mitigated through capturing stationary data in a calibration period, which can be used to normalize the signals from this sensor across participants. By selecting a ToF sensor over a more data-rich LiDAR camera present in other studies on terrain classification [23,24,25], the system cannot perform spatial mapping but rather aims to protect the privacy of users, and, through combining this sensor with the color sensor, this combination of sensors captures the equivalent data as a single pixel from these established LiDAR camera approaches. Variation in the distance between the sensor and the ground should aid in determining the gait cycle when considering the low-frequency data and in building a noise profile of the ground underfoot when considering the high-frequency data. These noise profiles can be used to separate terrains that are similarly colored but vary in texture. The LiDAR has a minimum operating distance of 40 mm [26], so it cannot be mounted with the color sensor and must be separately attached much higher up on the shank.

The combination of these sensor modalities should provide the system with comparable data to many recent HAR and terrain classification studies [7,12,13,14,15,16,17,19,20,21,23,24,25], incorporating an experimental combination of color sensor and LiDAR data to emulate a single pixel from a depth camera that retains the privacy of wearers whilst enabling the high accuracy that this sensor modality achieves in the literature.

Each sensor is encased in a small protective box and is connected via the I2C protocol to one of 3 Arduino Nano 33 BLE microprocessors (Arduino, Monza, Italy) positioned on the waist and each shank. These microprocessors perform the sampling and labeling of data, along with storing the data on an SD card. Samples are taken when the Arduino receives a command containing the activity number and time via Bluetooth from a central Arduino Mega. This approach synchronizes the time of all samples, along with allowing the system to be remotely controlled via a laptop using a custom serial interface.

Samples are captured at 16 ms intervals, resulting in a sample rate of 62.5 Hz. This value was determined through experimentation as higher sample rates resulted in the serial buffer of the Arduino Nano 33 BLE filling over time until data loss occurred.

**Figure 3:**
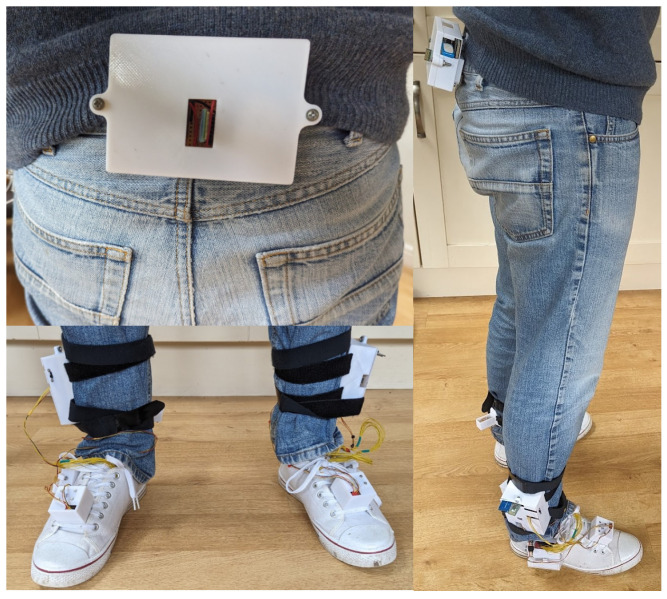
A subject equipped with the sensor system.

An image of the full sensor system when worn by a subject can be found in Figure 3.

### 2.2. Data Collection Procedure

**Figure 4:**
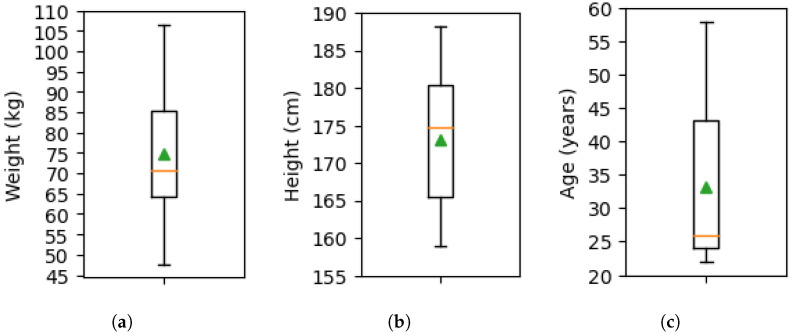
Box plots of demographic data for the 20 participants, along with the median (orange) and mean (green) values. (**a**) Weight; (**b**) height; (**c**) age.

Ethical approval was obtained from the University of Leeds with the reference MEEC 21-016 to recruit 20 healthy subjects (10 male and 10 female) from a pool of students and staff at the University of Leeds. Participants belonged to a wide range of ethnic backgrounds and had a mean weight of 74.69 ± 15.63 kg, height of 173.02 ± 8.71 cm, and age of 33.10 ± 11.34, as seen in Figure 4. Informed consent was collected from participants, after which they performed 38 tasks featuring 11 activities on 9 indoor and outdoor terrains. The chosen activities were walking, standing, ramp ascend, ramp descend, stair ascend, stair descend, sit-to-stand, stand-to-sit, sitting, going up in an elevator, and going down in an elevator. These activities were performed outdoors on grass, gravel, paving slabs, and asphalt, and indoors on laminated flooring in clinical rooms, hospital stairs, a bathroom, an elevator, and a carpeted floor in the hospital chapel. Activities and terrains were paired to cover the most common situations a person would encounter in daily life. The full list of activities and terrains can be found in Table 1. Data collection occurred at 11 am on Fridays between November 2022 and March 2024. As such, a range of ambient temperatures and weather conditions were captured throughout all seasons, reinforcing the real-environment aspect of this dataset.

Data were captured in order of participant number, except for participants 1, 13, and 3, who repeated their trials, in the specified order, after participant 20. On the first pass, participant 13’s outdoor data capture was canceled due to rain for multiple consecutive weeks until they became unavailable to participate. As such, they were substituted for another participant at the end of the trials. Participants 1 and 3 repeated their trials to improve the quality of the dataset as some data loss occurred in the earliest trials. In addition to capturing the core activities, participants 1 and 3 performed additional tasks during their repeat trials for the purpose of producing a test set, which can be used to verify the generalization capabilities of classifiers trained on the core dataset. These additional tasks introduce new combinations of existing activities and terrains and can be found in Table 2.

Due to the real-world nature of this dataset, environmental changes affected the data collection procedure, which resulted in changes in location due to availability, the splitting of sessions between 2 consecutive weeks due to rain or snow, or missing files due to damage to the sensor system and interference from a mobile Magnetic Resonance Imaging (MRI) machine temporarily located in the hospital car park. The most prominent of these changes was to the setup room where the laminated flooring activities were performed, which changed between room 1 (a meeting room), room 2 (a therapy room), and room 3 (the changing rooms of a hydrotherapy pool), along with the carpeted rooms that varied between the chapel and one of the offices on a ward. Additionally, whilst all participants were healthy and capable of independent walking, participant 15 disclosed that they had arthritis of the right foot and plantar fasciitis of the left foot. However, this participant still met the inclusion criteria for the study and completed the activities without issue.

### 2.3. Analysis

As this dataset is proposed for the purpose of HAR and terrain classification using machine learning, this study conducts an initial analysis into the dataset using various deep learning models. This builds on our previous work in which the dataset was analyzed with classical machine learning models such as Artificial Neural Networks (ANNs), Support Vector Machines (SVMs), K-Nearest Neighbors (KNN), and Random Forests (RFs), which achieved accuracies of up to 94% depending on the experimental setup [27,28]. In this study, we employ Convolutional Neural Network (CNN), Long Short-Term Memory (LSTM), and hybrid ConvLSTM architectures to evaluate the suitability of the dataset to deep learning applications, and explore the sensor modalities that contribute the most towards high classification accuracy.

**Figure 5:**
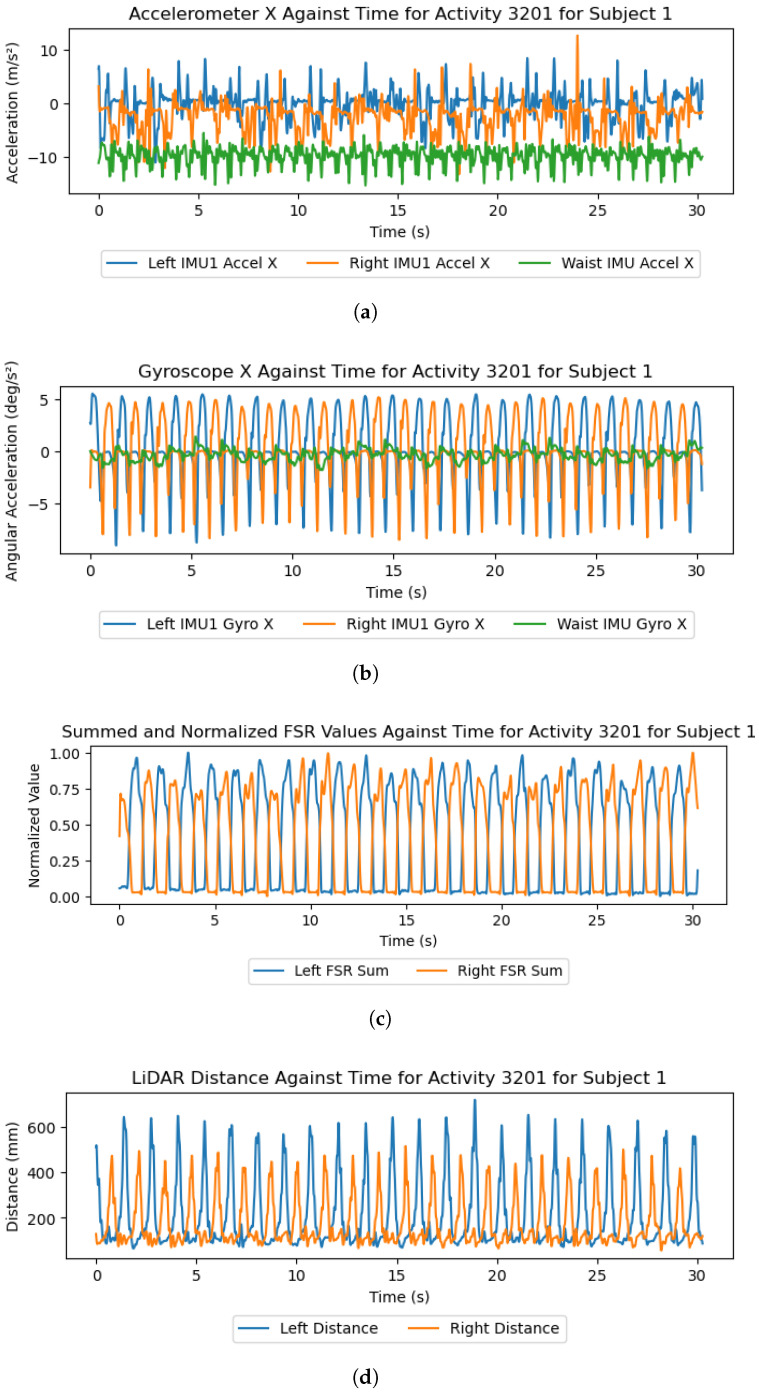
A selection of graphs that plot the data from the IMU X axis, summed and normalized FSR values, and LiDAR distances from subject 1’s “3201.txt” file, filtered using a 4th-order Butterworth low-pass filter with a 10 Hz cut-off frequency. (**a**) IMU accelerometer X axis; (**b**) IMU gyroscope X axis; (**c**) normalized FSR values; (**d**) LiDAR distance.

**Figure 6:**
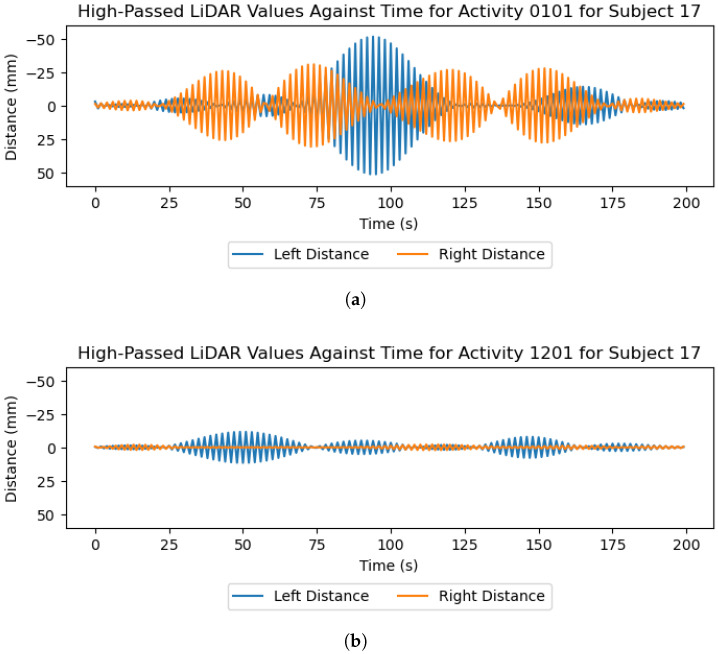
LiDAR readings high-pass-filtered at a cut-off frequency of 30 Hz for the walking activities on grass and laminated flooring for subject 17. (**a**) Grass; (**b**) laminated flooring.

To prepare the data for analysis, the included Python script (version 3.9.18) is used to reformat, merge, and label the raw data. As each of the feet and waist subsystems assigned common timestamps to samples, this initial stage merges these data on the shared timestamps and labels each sample with the activity, terrain, and activity–terrain combination referred to as the ’label’. Missing values are imputed using Scikit Learn’s IterativeImputer with 10 maximum iterations to ensure data integrity for trials affected by sensor errors. The data are then filtered using the SciPy implementation of a zero-phase Butterworth filter with differing filter parameters for each sensor type. The IMU data are filtered using a 4th-order low-pass filter with a cut-off frequency of 15 Hz, which is common among HAR studies using IMUs [29,30]. Data from the FSR insoles are filtered at 5 Hz, which is also common among studies using FSR insoles for gait-related applications [31,32]. Finally, data from the LiDAR are duplicated, with one copy being low-pass-filtered at 10 Hz and the other being high-pass-filtered at 30 Hz, which were determined experimentally and can be seen in Figure 5 and Figure 6.

After filtering, the raw data are partitioned into windows, each containing 2 s of data with an offset of 0.5 s between windows. To account for the lack of ’sit-to-stand’ and ’stand-to-sit’ data, window offsets are reduced to 0.125 s for these classes. Windows are then divided into a test set comprising 20% of the total windows and a training and validation set containing the remaining 80%. A scaler is then constructed using Scikit Learn’s MinMaxScaler function, which is fit to the train set and then applied to the test set to scale all values between 0 and 1 while preventing information leakage.

For classifying both activity and terrain, the task can either be treated as a single classification task using the activity–terrain combination as the sole label or through the decision-level fusion of classifications from two separate models that each individually predict activity and terrain. When using a combined label for activity and terrain, the potential classification space is reduced, resulting in higher accuracies at the cost of restricting the model to only the combinations featured in the train set. The architecture combining two individual classifiers, however, has the potential to predict activity–terrain combinations not present in the train set, provided that each individual model achieves sufficient accuracy and generalization. In this study, both architectures are implemented to evaluate the performance difference between each approach.

Three model architectures are evaluated for their performance on the CAHAR dataset, the feature extraction layers for which can be seen in Table 3. After these layers, each model features 512 densely connected neurons, a dropout layer with a dropout rate of 0.5, and the output layer.

To explore the contributions of each sensor modality to classification accuracy, an input perturbation analysis was conducted in which each sensor modality was obfuscated with noise to emulate its removal from the dataset. Gaussian noise was applied to each window of data as follows:

Let $x_{i,t,f}$ denote the value of feature *f* at window offset time *t* in window *i*, where i=1,…,N, t=1,…,T, and f=1,…,F.

Let M⊂{1,…,F} denote the set of features corresponding to a given sensor modality.

For obfuscation, all features belonging to the sensor modality M are replaced by Gaussian noise:

$${\tilde{x}}_{i,t,f}=\left\{\begin{array}{cc} N(\mu_{f},\sigma_{f}^{2}), & f\in M,\\ x_{i,t,f}, & f\notin M. \end{array}\right.$$

where

$$\mu_{f}=\frac{1}{NT}\sum_{i=1}^{N}\sum_{t=1}^{T}x_{i,t,f},\;\sigma_{f}^{2}=\frac{1}{NT}\sum_{i=1}^{N}\sum_{t=1}^{T}{\left(x_{i,t,f}-\mu_{f}\right)}^{2}.$$

The classification accuracy drop of the model can then be observed while each sensor modality is obfuscated to determine the importance of that sensor modality through its contribution towards the overall classification accuracy.

## 3. Results

### 3.1. Dataset Collection and Visualization

From each of the 20 participants, 85–86 activity files are captured per leg, with a less consistent amount from the waist, resulting in a dataset of 4865 activity files containing over 7.8 h of gait data. This data is stored in text files with the format “XXYY.txt”, with the former digits referring to the activity index found in Table 1 and Table 2, whilst the latter digits refer to the trial number for that activity. Walking trials were repeated twice, with the summary digits “01” for counterclockwise and “02” for clockwise turning. The exception to this was with the gravel and paving slab activities, which took place on a linear walkway. As such, these trials were repeated three times, with each repeat containing one full length of the walkway. Sit-to-stand, stand-to-sit, stair, and ramp activities were each repeated three times, whilst standing, sitting, and the elevator activities contain only one trial each. The total time spent performing each activity and on each terrain in the dataset can be seen in Table 4 and Table 5, while a summary of the CAHAR dataset’s composition in comparison to related works is detailed in Table 6.

For the leg subsystems, each file contains the following data channels: time elapsed in milliseconds, LiDAR distance in millimeters, each of the nine-axis IMU channels, unprocessed light value ’C’, red, green, and blue color values in the range 0-255, color temperature, processed light value ’Lux’, and the 13 FSR values in the range 0-1023. The waist sensor contains only the time elapsed and IMU channels in the same format. Example data readings from the accelerometer, gyroscope, FSR insoles, and LiDAR sensors can be found in Figure 5.

**Figure 7:**
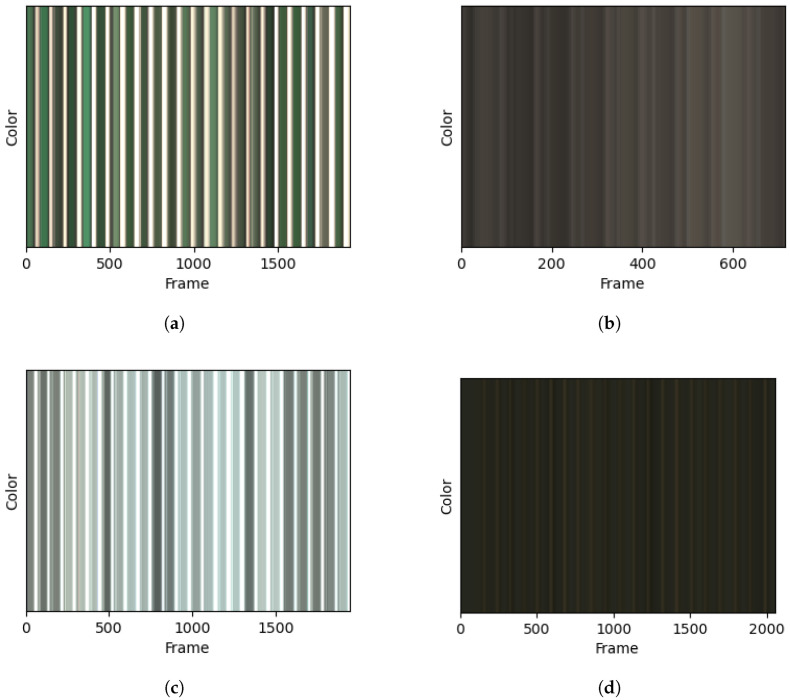
Color over time captured from the left leg during walking on 4 different flooring types. (**a**) Grass; (**b**) gravel; (**c**) asphalt; (**d**) laminated flooring.

Figure 7 shows the combined red, green, and blue channels from the color sensor over time for four of the walking trials. These readings reflect the color of the floor underfoot, along with the ambient light level that is visible when the foot is lifted from the ground. The indoor and gravel activities were captured in the shade, resulting in dark ambient light levels during the foot-off segments, whereas the outdoor grass and asphalt trials demonstrate the capture of white ambient light between strides.

Regarding the coarseness of each terrain, removing the low-frequency LiDAR information pertaining to individual strides, as seen in Figure 5d, reveals that the high-frequency information reflects the frequency and magnitude of height fluctuations in the terrain underfoot. Figure 6 shows these high-frequency LiDAR readings, which demonstrate that the coarse grass terrain can be separated from the smoother laminated flooring.

**Figure 8:**
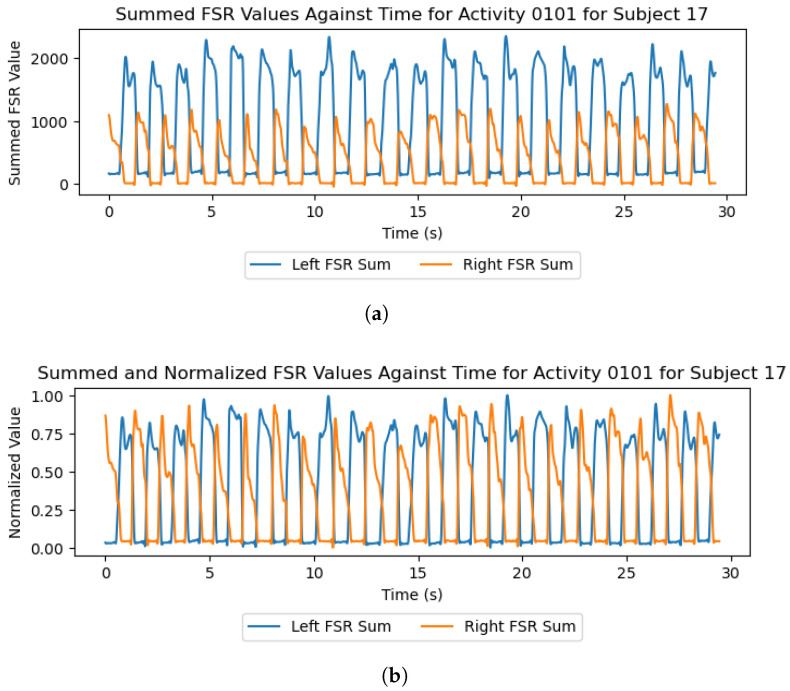
Plots of the insole data from the walking-on-grass trial of participant 17 when summed and normalized. The data is filtered using a 4th-order Butterworth low-pass filter with a 10 Hz cut-off frequency. (**a**) Summed; (**b**) summed and normalized.

The trials feature varying qualities of insole data depending on the version of the insole and the number of participants who had previously used that insole. Examples of this can be found in Figure 8a, where the left-foot insole values rest at a higher level than the right insole values. This issue is more prevalent in the version 1 insoles and resulted in data from some sensors being offset by a given value. This can be mitigated through the normalization of FSR values, as seen in Figure 8b, or by using the standing trials and participant weight to calibrate the sensors if exact force values are required.

**Figure 9:**
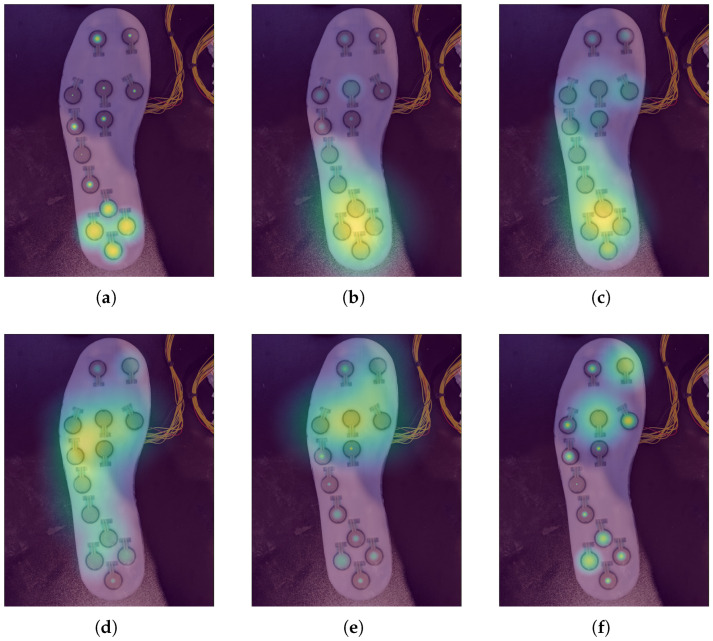
Heat maps of the FSR data 160 ms apart during subject 1’s walking-on-carpet trial. (**a**) 480 ms; (**b**) 640 ms; (**c**) 800 ms; (**d**) 960 ms; (**e**) 1120 ms; (**f**) 1280 ms.

Tools are included with the dataset that perform data cleaning and reformatting, along with generating animated heat maps of the force on each FSR for a given activity. These heat maps allow for the visualization of force distributions of each foot for any given activity. Example frames from one of these heat maps for subject 1’s walking-on-carpet trial can be found in Figure 9.

### 3.2. Analysis

The test accuracy results of the CNN, LSTM, and ConvLSTM architectures are found in Table 7, Table 8 and Table 9, respectively. Of these three architectures, the CNN achieves the highest performance metrics for the activity and terrain classification, resulting in the highest activity–terrain decision fusion accuracy, while LSTM exhibits the highest accuracy when classifying using the fixed 38 labels.

Input perturbation was performed for the CNN only due to its high performance compared to the LSTM and ConvLSTM. The accuracy and accuracy drop of each model with each sensor modality obfuscated can be found in Table 10 and Table 11, respectively. From these results, the IMU and color sensor are highlighted as modalities with the greatest impact across all classification tasks, while LiDAR is primarily relevant for activity classification, and the FSR insole contributes more towards terrain classification.

## 4. Discussion

Recent successes in high-accuracy HAR and the calculation of spatio-temporal parameters using wearable sensors indicate that remote gait analysis is an accessible technology following the accurate labeling of collected data with contextual information such as activity and terrain. Data collected from remote gait analysis cannot be reliably used to aid in the diagnosis or prognosis of gait-affecting conditions unless this contextual information about the collected data is present due to the dependency of gait on the terrain underfoot [10,11]. Whilst some datasets have been proposed to address this issue in recent years [12,13], many of the core activity and terrain combinations required to enable real-world remote gait analysis are not present, and a balance is lacking between indoor and outdoor activities. We propose a sensor system comprising both established and unexplored sensor types in the fields of HAR and gait analysis to collect the first terrain-labeled HAR dataset featuring a range of walking activities performed on both indoor and outdoor terrains.

A comparison of the dataset in this study to existing real-world datasets can be found in Table 6, which highlights the increased number of activity–terrain combinations (walking contexts) included in this dataset. Luo et al. [12] focus on walking activities, with walking-, ramp-, and stair-based activities being performed on seven outdoor terrains. In this dataset, a single activity is performed on each terrain, apart from the stair and ramp activities, which are split by ascent and descent on the same terrain. Thirty participants then perform these tasks whilst equipped with six IMUs. This dataset is suited to studying gait mechanics on different terrains but lacks the full suite of indoor terrains and, by not repeating different activity types on the same terrain, introduces overfitting risks when considering classification models. Stairs induce significant gait changes that make such an activity easily distinguishable from level-ground walking. If stair ascent and descent are performed on a single terrain, and no other activities are performed on that terrain, then classification models can be expected to classify that terrain with high accuracy, which impacts the generalization of models built from such a dataset. Losing and Hasenjäger [13] take some measures to avoid this, with 12 combinations of nine activities and five terrains resulting in several walking and turning activities taking place on the same terrain. However, similar to the dataset by Luo et al. [12], repetitions of walking activities with significantly different gait signatures, such as walking and stair ascent/descent, are not performed on the same terrains. Furthermore, both datasets do not include activities such as sit-to-stand/stand-to-sit and stationary standing and sitting. By including the wide range of 38 combinations of 11 activities and nine terrains in this study, the CAHAR dataset is more suited to the development of machine learning classification models than existing datasets and provides the necessary activity and terrain labeling, as well as a dedicated test set of nine unseen combinations, that enable researchers to build generalized models suited to real-world deployment and capable of high accuracy in unsupervised free-living settings.

The results and preliminary investigations into the captured data demonstrate the multidisciplinary capabilities of this dataset. IMUs and FSR insoles are known to be powerful sensors in the areas of HAR [12,13] and gait-phase detection [4,6,33], with Figure 5 and Figure 9 demonstrating the capability of the proposed system in these areas. Regarding the terrain-specific aspects of the dataset, Figure 6 and Figure 7 show the effectiveness of the color and LiDAR sensors in extracting the color and coarseness of the terrain underfoot. Furthermore, Figure 5d shows clear peaks in the LiDAR data where the swing phase occurs when aligned with the FSR data. In addition, the color maps in Figure 7 show that the color sensor captures the ambient light when the foot is lifted. As such, it is likely that some activities, such as walking and standing, could be determined with LiDAR and color sensor data alone, which may help to reduce the profile of the proposed sensor system in future studies. However, Figure 7 also highlights the performance of the color sensor in different light levels, which may affect the utility of this sensor. To mitigate this, data capture occurred intermittently over 17 months to ensure that a wide variety of light levels and ambient conditions were captured, although data were captured in daylight hours only. The TCS34725 color sensor features a built-in Light-Emitting Diode (LED) to accommodate low-light-level environments [22]; however, this does not seem to have been effective at enabling the sensor to accurately capture the color of the laminated flooring, which should have been blue. Additionally, while the sensor’s proximity limitations were met through the attachment on the side of the shoe, other limitations like vulnerability to shadows and ambient light levels may introduce a lack of generalization when comparing the indoor environments in this dataset to other similar indoor environments. It is recommended to use consistent indoor lighting conditions to normalize the color sensor readings across participants.

Regarding the analysis of the CAHAR dataset using deep learning architectures, CNN and LSTM exhibit similar performances, with CNN achieving a higher classification accuracy on the individual activity and terrain classifiers, while LSTM performs best when considering each activity–terrain combination as a separate label. Both models outperform the hybrid ConvLSTM model, which may indicate that, in this case, the implementation of this architecture caused overfitting due to its size and complexity. When compared with our previous analysis using classical machine learning models, the deep learning models perform similarly when considering the 38 combinations of activity and terrain as labels, with an accuracy of 94% in both cases. However, the accuracy of the multi-model approach utilizing decision fusion was significantly higher across all the deep learning models, with the CNN achieving 93% accuracy compared with the 85% accuracy of the SVM—the highest-performing classical machine learning model [27]. The input perturbation analysis also demonstrates agreement with our previous analysis, in which the IMU and color sensor appeared as the sensor modalities that contribute the most towards high classification accuracy [28]. For the deep learning models, the color sensor and IMU modalities were generally the largest contributors towards classification accuracy, with LiDAR data being more important for activity classification and FSR data contributing more to terrain classification. This may indicate that the FSRs reliably respond to the coarseness of the terrain underfoot through the weight distribution of the foot, while the LiDAR modality appears to be better suited to activity than terrain and may need additional preprocessing to highlight the differences seen in Figure 6. While informative for situations in which the subject data is included in the train set, future work should aim to explore the performance of models under the circumstances that data from a whole subject is withheld from training and used for testing. This approach increases the generalization requirements and typically results in lower accuracies but works towards developing analytical methods for ’off-the-shelf’ activity and terrain classification that are more suited to real-world implementation. These preliminary findings demonstrate the potential for future researchers to investigate novel sensor types for the purpose of optimizing wearable gait analysis systems, along with applying powerful classification techniques capable of accurate terrain and activity classification, to bring remote gait analysis closer to implementation in healthcare settings.

An additional factor that may affect the performance of classifiers built from this dataset is the inherent relationship between activities and terrains. For example, models are unlikely to predict activity–terrain combinations that are not present in the training set, such as sit-to-stand on grass. This may be further emphasized by the fixed lighting levels, sensor noise, and presence of shadows on the terrains in this dataset, which may reduce the generalization capabilities of models built from this dataset. While some of these combinations are rare in real-world scenarios, such as the presence of stairs in a bathroom, the extra activities captured and shown in Table 2 can be used to verify that models can classify activity and terrain independently and beyond the limited combinations of the main dataset. Data augmentation approaches may help to mitigate these terrain- and instance-specific factors and improve model generalization.

Although this dataset offers a large novel selection of activities and terrains, limitations are still present in this study, primarily sourced from the damage to the sensor system throughout the trials. This issue was mitigated via two-way communications between the Arduino Mega and Arduino Nano 33 BLE sensors, ensuring that no data was lost from either of the leg subsystems. However, much of the waist sensor data was corrupted in the first 12 trials due to these issues. Another limitation of this dataset is related to the sit-to-stand and stand-to-sit activities. These activities are extremely quick to perform, resulting in fewer data from these trials than others, such as the 30 s of walking, which could lead to large class imbalances when performing classification. This was mitigated by asking participants to repeat this trial three times, but, in the future, activities should be performed for equal times to ensure that classification models have sufficient data to train on. Additionally, the use of scripted predefined walking routes may influence the behavior of participants, particularly in comparison with the free-living gait data that models built with this dataset should be able to classify with high accuracy. Subjects in this study perform a single walking task with a stationary period at the start and end, meaning that transitionary states and variability in gait speed are minimized. Whilst appropriate for this initial exploration into privacy-retaining context-aware activity recognition, future work should aim to deploy this sensor system in a semi-supervised context to expand the dataset with free-living data.

## 5. Conclusions

This study proposes a novel HAR dataset composed of 38 combinations of 11 walking activities performed on nine terrains by 20 healthy subjects, totaling 7.8 h of continuous activity data. As the first HAR dataset to label activity and terrain separately, this dataset enables new developments in determining the full context surrounding a person’s gait data to enable the design of analytical techniques required for implementing remote gait analysis using wearable sensors. A novel sensor system is designed to collect this dataset, which combines popular proven HAR and gait analysis sensors, such as IMUs and force-sensing insoles, with previously unexplored color and LiDAR sensors, which are shown to be capable of determining properties such as the color and coarseness of the terrain underfoot. This sensor system produces a rich dataset that is suited for analysis with powerful deep learning techniques to attain the high accuracy, precision, and generalizability needed to enable the adoption of context-aware remote gait analysis technologies in healthcare settings.

Future work in this area will include the augmentation of this dataset with data collected from people with gait-affecting conditions, such as Parkinson’s disease and dementia. In addition, a context-aware remote gait analysis system will be developed to reduce the healthcare burden of gait analysis and increase the capabilities for diagnosis and prognosis in gait-affecting conditions.

## Figures and Tables

**Table 1:** The full activity list for data collection.

Index	Activity	Terrain	Exercise
1	Walking	Grass	2×—Walk for 30 s
2	Ramp Ascend	Grass	3×—Walk up the ramp
3	Ramp Descend	Grass	3×—Walk down the ramp
4	Standing	Grass	1×—Stand still for 10 s
5	Walking	Paving Slabs	3×—Walk for 10 s
6	Walking	Gravel	3×—Walk for 10 s
7	Standing	Paving Slabs	1×—Stand still for 10 s
8	Standing	Gravel	1×—Stand still for 10 s
9	Sit-to-Stand	Paving Slabs	3×—Stand up from a chair
10	Stand-to-Sit	Paving Slabs	3×—Sit down on a chair
11	Sitting	Paving Slabs	1×—Stay sitting for 10 s
12	Walking	Laminated Floor	2×—Walk for 30 s
13	Standing	Laminated Floor	1×—Stand still for 10 s
14	Sit-to-Stand	Laminated Floor	3×—Stand up from a chair
15	Stand-to-Sit	Laminated Floor	3×—Sit down on a chair
16	Sitting	Laminated Floor	1×—Stay sitting for 10 s
17	Stair Ascend	Laminated Floor	3×—Walk up a set of stairs
18	Stair Descend	Laminated Floor	3×—Walk down a set of stairs
19	Sit-to-Stand	Bathroom	3×—Stand up from a chair
20	Stand-to-Sit	Bathroom	3×—Sit down on a chair
21	Sitting	Bathroom	1×—Stay sitting for 10 s
22	Stair Ascend	Paving Slabs	3×—Walk up a set of stairs
23	Stair Descend	Paving Slabs	3×—Walk down a set of stairs
24	Ramp Ascend	Paving Slabs	3×—Walk up the ramp
25	Ramp Descend	Paving Slabs	3×—Walk down the ramp
26	Ramp Ascend	Asphalt	3×—Walk up the ramp
27	Ramp Descend	Asphalt	3×—Walk down the ramp
28	Walking	Asphalt	2×—Walk for 30 s
29	Standing	Asphalt	1×—Stand still for 10 s
30	Elevator Down	Elevator	1×—Travel down in the elevator
31	Elevator Up	Elevator	1×—Travel up in the elevator
32	Walking	Carpet	2×—Walk for 30 s
33	Standing	Carpet	1×—Stand still for 10 s
34	Sit-to-Stand	Carpet	3×—Stand up from a chair
35	Stand-to-Sit	Carpet	3×—Sit down on a chair
36	Sitting	Carpet	1×—Stay sitting for 10 s
37	Stair Ascend	Hospital Stairs	3×—Walk up a set of stairs
38	Stair Descend	Hospital Stairs	3×—Walk down a set of stairs

**Table 2:** The additional activity list for subjects 1 and 3.

Index	Activity	Terrain	Exercise
39	Sit-to-Stand	Gravel	3×—Stand up from a chair
40	Stand-to-Sit	Gravel	3×—Sit down on a chair
41	Sitting	Gravel	1×—Sit still for 10 s
42	Sit-to-Stand	Grass	3×—Stand up from a chair
43	Stand-to-Sit	Grass	3×—Sit down on a chair
44	Sitting	Grass	3×—Sit still for 10 s
45	Standing	Bathroom	1×—Stand still for 10 s
46	Ramp Ascend	Laminated Floor	3×—Walk up the ramp
47	Ramp Descend	Laminated Floor	3×—Walk down the ramp

**Table 3:** Summary of feature extraction layers in each model architecture.

Architecture	Layer 1	Layer 2	Layer 3	Layer 4	Layer 5
CNN	Conv1D (128)	Max Pooling	Conv1D (256)	Global Average Pooling	–
LSTM	LSTM (256)	Dense (256)	–	–	–
ConvLSTM	Conv1D (128)	Max Pooling	Conv1D (256)	Max Pooling	LSTM (256)

**Table 4:** Total duration of recorded data per activity.

Activity	Duration (min)
Walking	182.02
Ramp Ascend	56.14
Ramp Descend	55.40
Standing	41.77
Sit-to-Stand	18.70
Stand-to-Sit	21.39
Sitting	28.19
Stair Ascend	22.58
Stair Descend	21.75
Elevator Down	11.56
Elevator Up	11.31

**Table 5:** Total duration of recorded data on each terrain.

Terrain	Duration (min)
Grass	78.20
Paving Slabs	87.72
Gravel	30.71
Laminated Flooring	72.17
Bathroom	17.93
Asphalt	75.20
Elevator	22.87
Carpet	59.27
Hospital Stairs	26.75

**Table 6:** Real-terrain activity recognition datasets.

Dataset	Subjects	Sensors	Activities	Terrains	Activity–Terrain Combinations	Locations
Luo et al. [12]	30	6× IMUs	5	7	9	9 outdoor, 0 indoor
Losing and Hasenjäger [13]	20	17× IMUs and barometer nodes, 2× insoles with 8x FSRs each, 1× eye tracker	9	5	12	12 outdoor, 0 indoor
Proposed CAHAR Dataset	20	3× IMUs, 2× LiDARs, 2× color sensors, 2× insoles with 13× FSRs each	11	9	38 + 9	19 outdoor, 19 indoor

**Table 7:** Evaluation metric summary for the CNN architecture when predicting activity, terrain, label (combined activity and terrain), and when using a decision fusion of the individual activity and terrain classifications.

Task	Accuracy	Sensitivity (Recall)	Specificity	F1 Score
Activity	0.9467	0.9199	0.9943	0.9238
Terrain	0.9865	0.9873	0.9981	0.9833
Label	0.9395	0.9219	0.9983	0.9261
Fusion	0.9349	0.7245	0.9982	0.7254

**Table 8:** Evaluation metric summary for the LSTM architecture when predicting activity, terrain, label (combined activity and terrain), and when using a decision fusion of the individual activity and terrain classifications.

Task	Accuracy	Sensitivity (Recall)	Specificity	F1 Score
Activity	0.9379	0.9122	0.9936	0.9071
Terrain	0.9827	0.9818	0.9975	0.9797
Label	0.9443	0.9352	0.9984	0.9354
Fusion	0.9244	0.6044	0.9980	0.6073

**Table 9:** Classification performance metrics for the ConvLSTM architecture when predicting activity, terrain, label (combined activity and terrain), and when using a decision fusion of the individual activity and terrain classifications.

Task	Accuracy	Sensitivity (Recall)	Specificity	F1 Score
Activity	0.9374	0.8793	0.9931	0.8786
Terrain	0.9791	0.9797	0.9970	0.9752
Label	0.9183	0.8983	0.9977	0.9007
Fusion	0.9201	0.6008	0.9979	0.6043

**Table 10:** Classification accuracy of each model after obfuscation of each sensor modality.

Modality	Activity	Terrain	Label
Baseline	0.9467	0.9865	0.9395
IMU	0.0652	0.2518	0.0163
FSR	0.3823	0.1531	0.1063
LiDAR	0.1764	0.4092	0.1901
Color sensor	0.1742	0.1830	0.0467

**Table 11:** Classification accuracy drop (Δ accuracy) when each sensor modality is obfuscated.

Modality	Activity	Terrain	Label
Baseline	0.0000	0.0000	0.0000
IMU	0.8815	0.7346	0.9231
FSR	0.5644	0.8334	0.8332
LiDAR	0.7703	0.5773	0.7494
Color sensor	0.7725	0.8035	0.8928

## Data Availability

The original data presented in the study are openly available in IEEE DataPort at DOI 10.21227/bwee-bv18.

## Abbreviations

The following abbreviations are used in this manuscript:

ANN	Artificial Neural Network
CAHAR	Context-Aware Human Activity Recognition
CNN	Convolutional Neural Network
CoP	Center of Pressure
EMG	Electromyography
FSR	Force-Sensing Resistor
HAR	Human Activity Recognition
IMU	Inertial Measurement Unit
IoT	Internet of Things
KNN	K-Nearest Neighbors
LED	Light-Emitting Diode
LSTM	Long Short-Term Memory
MRI	Magnetic Resonance Imaging
PLA	Polylactic Acid
RF	Random Forest
SVM	Support Vector Machine
ToF	Time-of-Flight
UI	User Interface
WHO	World Health Organization
